# RintC: fast and accuracy-aware decomposition of distributions of RNA secondary structures with extended logsumexp

**DOI:** 10.1186/s12859-020-3535-5

**Published:** 2020-05-24

**Authors:** Hiroki Takizawa, Junichi Iwakiri, Kiyoshi Asai

**Affiliations:** 1 Graduate School of Frontier Sciences, The University of Tokyo, Chiba, Japan; 2 Artificial Intelligence Research Center (AIRC), National Institute of Advanced Industrial Science and Technology (AIST), Tokyo, Japan

**Keywords:** RNA secondary structure, Dynamic programming, Accuracy-guaranteed numerical computation, Interval arithmetic, Ribosomal RNA.

## Abstract

**Background:**

Analysis of secondary structures is essential for understanding the functions of RNAs. Because RNA molecules thermally fluctuate, it is necessary to analyze the probability distributions of their secondary structures. Existing methods, however, are not applicable to long RNAs owing to their high computational complexity. Additionally, previous research has suffered from two numerical difficulties: overflow and significant numerical errors.

**Result:**

In this research, we reduced the computational complexity of calculating the landscape of the probability distribution of secondary structures by introducing a maximum-span constraint. In addition, we resolved numerical computation problems through two techniques: extended logsumexp and accuracy-guaranteed numerical computation. We analyzed the stability of the secondary structures of 16S ribosomal RNAs at various temperatures without overflow. The results obtained are consistent with previous research on thermophilic bacteria, suggesting that our method is applicable in thermal stability analysis. Furthermore, we quantitatively assessed numerical stability using our method..

**Conclusion:**

These results demonstrate that the proposed method is applicable to long RNAs..

## Background

Functional non-coding RNAs (ncRNAs) play essential roles in a wide range of biological phenomena. Secondary structures are often crucial to the functions of RNAs. A number of studies and software tools can predict a single secondary structure for a given RNA sequence [1]. According to detailed analyses of free energy, however, some RNAs do not always form a single stable structure. Therefore, quantitative evaluations of the fluctuation of RNA secondary structures have recently attracted attention. Recent studies have provided methods to analyze the distribution of RNA secondary structures in more detail using the marginal probability of Hamming Distance, in which each RNA structure is located in a discrete metric space [2–4].

Structures of long ncRNAs (*e.g.*, >1000 bases, including ribosomal RNAs) are important for understanding their functions, but analyzing the probability landscape of the structure remains a challenging task. Fourier transform has been utilized to reduce computational complexity [5–7], but the computational costs of previous methods are still too high to apply to long ncRNAs. Furthermore, the Fourier transform distributes numerical errors uniformly across large and small marginal probabilities, which makes small marginal probabilities unreliable.

Small marginal probabilities, however, are also of interest occasionally. In kinetic analyses, for example, meta-stable structures may have considerably higher free energy compared to the minimum free energy structure [8]. In such a case, the Boltzmann probability of the meta-stable regions can be very small. For reliable evaluation, quantitative assessment of numerical errors is necessary. Previous studies have described this type of numerical instability, but they have not shown detailed analyses [9].

To provide quantitative evaluation of numerical instability, we have implemented accuracy-guaranteed numerical computation based on interval arithmetic and evaluated the numerical errors associated with the Fourier transform. Interval arithmetic is a method in which arithmetic operations are defined along intervals expressing numerical values between the upper/lower edges. The approximate calculation of pi by Archimedes in the 3rd century BC is known as the oldest example of interval arithmetic. Around the 1950s, interval arithmetic came to be used for estimating the upper bounds on the numerical error caused by floating-point arithmetic in computers. For example, Sunaga [10] published one of the first studies in English on comprehensive algorithms for interval arithmetic for computers. Interval arithmetic for accuracy-guaranteed numerical computation has been established as a research field and detailed in a textbook [11].

To reduce computational costs, we have introduced the maximum-span constraint, which forbids long-range base-pairs. Such a constraint has been used for the prediction of secondary structures [12], but it has not been used to estimate marginal probabilities of discrete metrics (*e.g.*, Hamming distances). It may seem inappropriate to ignore long-range interactions in secondary structures because there are long-range interacting base-pairs in the 3D structures of long RNAs (*e.g.*, 16S rRNA). The predictions of long-range interactions, however, are known to be unreliable even if long-range base-pairs are allowed [13], while the widely used nearest neighbor energy model is not compatible with long RNAs and its parameters have been determined by experiments using short RNAs [14]. Accordingly, our method lost little by excluding long-range base-pairs. At the same time, we show that estimated stability based on our tool with maximum-span constraint was consistent with the previous research.

Maximum-span constraint has enabled the calculation of marginal probabilities on a discrete metric for long RNA sequences, but we had to cope with numerical overflow for calculations with long sequences. In stochastic models such as hidden Markov models and stochastic context free grammars, which are common for modeling and analyzing RNA structures, logsumexp (logarithm of the sum of exponentials) is the standard solution for preventing overflow or underflow in numerical calculation [15, 16]. There is a limitation, however, in that it cannot handle zero or negative values. This limitation is a problem when processing complex numbers with rectangular coordinates in the Fourier transform. One solution is to apply logsumexp only to radii using polar coordinates, but simple application of polar coordinates causes problems when combined with interval arithmetic for accuracy-guaranteed numerical computation. Complicated conditions occur when the angular interval crosses zero or the radius interval contains zero. In this paper, in addition to a radius of polar coordinates, normalized orthogonal coordinates, rather than angles, are combined for interval arithmetic of logsumexp. Consequently, we have realized the advantages of logsumexp and interval arithmetic while preserving the simplicity of implementation.

## Results

### Computation time

To demonstrate computational efficiency, the computation time of the proposed method using the S151 Rfam Dataset [15] was measured. In the proposed method, the reference structures were obtained by CentroidFold [17](*γ*=1.0). All cores of the Intel Core i7 4770 CPU were used as a computational resource in this measurement.

**Fig. 1:**
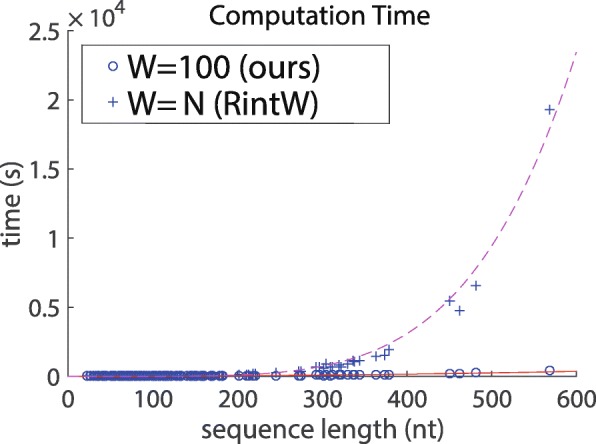
Calculation time of the proposed method. The red solid line represents *y*=0.0010101*x*^2^. The purple dashed line represents *y*=3.0163*e*−10*x*^5^. Both lines were fit to the result using the least squares method

We measured the computation time in the case where the maximum-span constraint *W*=100 is introduced and in the case where no restriction is applied (equivalent to RintW [7]) (Fig. 1).

**Fig. 2:**
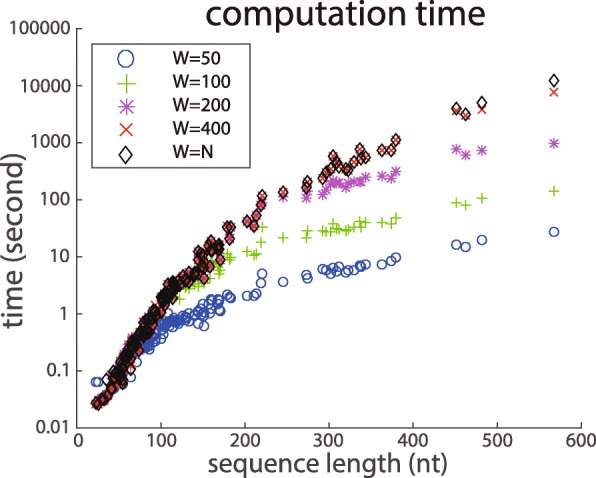
Calculation time of the proposed method. Each data point is the calculation time of a single sequence from the S151Rfam dataset. The *y*-axis represents the logarithm of calculation time, and the *x*-axis represents the length of RNA

We also examined how the calculation time changes when the value of the maximum-span constraint *W* is changed (Fig. 2). In this experiment, 32 Intel Xeon Gold 6130 cores were used for computation.

### Thermal stability of ribosomal RNA

As an application of the proposed method, we analyzed the thermal stability of the secondary structures of 16S rRNAs derived from *E. coli* and *T. thermophilus* using Credibility Limit [18] as the metric.

**Fig. 3:**
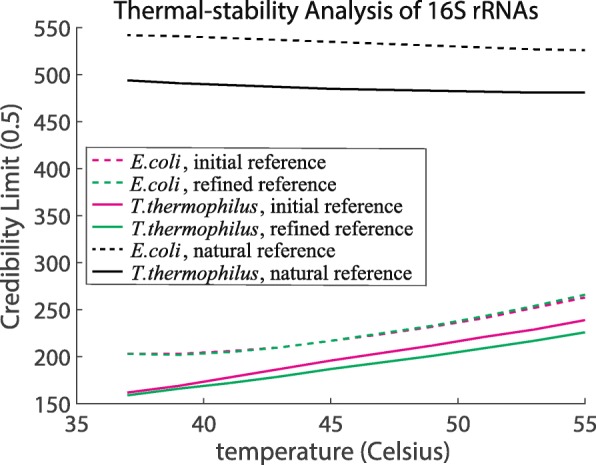
Thermal-stability analysis for secondary structures of *E. coli* and *T. thermophilus* 16S rRNAs. The "initial reference" is the reference structure obtained using CentroidFold. The "refined reference" is the reference structure obtained using RintC and the base-pairing probability matrix (BPPM) (see the “Thermal stability of ribosomal RNA” section for the details). The "natural reference" is the reference structure derived from the three-dimensional structure. The “Experimental procedure” section provides a detailed description of the "natural reference"

The Credibility Limit (0.5) of a given secondary structure was obtained with temperatures ranging from 37 to 55 degrees Celsius (Fig. 3). As the origin of the Hamming distance, three types of reference structure were prepared. (i) The "initial reference" was obtained using CentroidFold. (ii) The "refined reference" was obtained by the following steps: RintC was performed with the initial reference; a Hamming distance interval in which the probability is locally high was chosen; the BPPM of the interval was calculated; and a "refined reference" was obtained from the BPPM by posterior decoding with CentroidFold. (iii) The "natural reference" was the reference structure derived from the three-dimensional structure in NDB.

### Numerical error evaluation

**Fig. 4:**
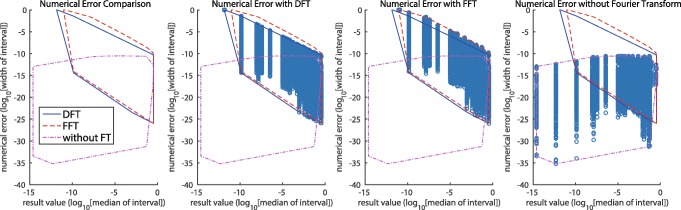
The result of the numerical error experiment with RF00008B (54 nucleotides). The leftmost plot explains the convex hulls for the result values and their errors under each experimental condition. The three plots to the right are scatter plots of the raw data for the result values and errors under one experimental condition and the convex hulls for each. In this evaluation, the reference structure was obtained by CentroidFold

For a quantitative evaluation of RintC numerical error, accuracy-guaranteed numerical computations with interval arithmetic were applied to the calculation process of RintC with the RF00008B sequence in the S151 Rfam dataset [15]. The length of the RF00008B sequence was short enough for the evaluation of time-consuming calculation without any type of Fourier transform. The numerical errors of three types of calculation (DFT, FFT, and non-FFT) are shown in Fig. 4.

**Fig. 5:**
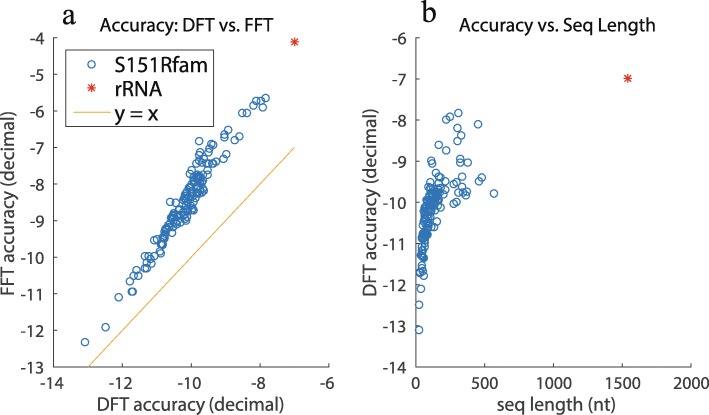
The results of the numerical error experiment. **a** Numerical error comparison. **b** Relationship between sequence length and numerical error. The *x*- and *y*-axes have minima of (*log*_10_(*median*)+*log*_10_(*width*)) under the DFT and FFT methods. The reference structure was obtained using CentroidFold [17]. (*γ*=1.0)

Numerical error evaluation was also conducted for all sequences in the S151 Rfam dataset and *E. coli* 16S rRNA (Fig. 5). Each data point corresponds to an individual sequence in the S151 Rfam dataset or *E. coli* 16S rRNA. In comparisons of the numerical errors between DFT and FFT versions (Fig. 5a), DFT is always more accurate than FFT. This result is consistent with that shown in Fig. 4. In addition, a relationship between the numerical error and sequence length in the DFT results was also investigated (Fig. 5b).

## Discussion

### Computational efficiency

Figure 1 shows that the computation time when using the constraint *W*=100 follows the theoretical complexities of the square of the length of the sequence, while computational time scales with the fifth power of the length of the sequence when no constraint is used. This confirms that computational complexity is drastically reduced by introducing the maximum-span constraint into the proposed method.

As Fig. 2 shows, when *W*<*N*, the calculation time is reduced by the effect of the maximum-span. When *N*≤*W*, the same calculation time is required regardless of the value of *W*. Since many of the data points were short RNAs, the differences for large W values were unclear. Nevertheless, the relationship between W and computation time was consistent with the theory. In the next subsection, we demonstrate that the proposed method works for long RNAs.

### Thermal stability of ribosomal RNA

The credibility limits of natural references are much higher than those of others, because natural references include long-range base-pairs. Maximum-span constraint *W*=100 was introduced because most of the actual spans of base-pairs are less than or equal to 100 bases (Supplementary Table S1), but long-range interactions may play an important role in structures. Thermophilic bacteria have reduced dynamics of intracellular macromolecules (mainly proteins) compared with mesophilic bacteria [19]. Several thermophilic RNAs exhibit higher thermal stability than the mesophilic homologous [20, 21]. In addition, thermal adaptation of the thermophilic ribosomal subunit including 16S rRNA has been suggested by structural and evolutionary analysis [22]. Our result using maximum-span constraint *W*=100 shows that the 16S rRNA of *T. thermophilus* had a lower Credibility Limit than that of E. coli, which also implies not only the protein components but also the rRNA playing a role in the thermal stability of thermophilic ribosomes (Fig. 3).

The use of the natural secondary structure as a representative structure exhibited a relatively higher Credibility Limit, compared with the "initial" and "refined" references. This implies the DP calculation with the Turner model is compatible for the representative structure derived from the Turner model, such as the "initial" and "refined" references. Note that this result would not indicate the advantage of "initial" and "refined" structures over the "natural" structures.

### Numerical error evaluation

As Fig. 4 shows, for the DFT and FFT methods, the numerical error (i.e., interval width) is almost equal to 1, when the calculated existence probability is quite small. Interval width =1 indicates that the probability is within [0,1], thus providing no meaningful information owing to the numerical error. In contrast, the numerical error remains low when the existence probability is moderate or high. DFT-based results are slightly more accurate than FFT-based results. In further numerical error comparisons between non-Fourier transform results and DFT or FFT results, the numerical error of the non-Fourier result is smaller than those of the DFT or FFT results. This implies that the problematic numerical error is indeed caused by Fourier transform.

Figure 5b demonstrates that the numerical error in the marginal probability of the structures for long RNA sequences (≥1000 nt) is sufficiently small (about 10^−7^ for 16S rRNA) for the structures with a moderate or high probability of existence. This accuracy is sufficient for thermal stability analysis because an accurate evaluation of large clusters is only required for their analysis.

## Conclusions

Since RNA secondary structures have large thermal fluctuations, prediction of the most stable secondary structure is insufficient for representing native structural behavior of an RNA molecule. Marginal probabilities on Hamming distances from reference structures, which represent the landscape of all the possible RNA secondary structures, can be efficiently computed by combining Fourier transform with dynamic programming, but the computational costs are still too high for long RNAs.

In this research, we have implemented a maximum-span constraint of base-pairs to reduce computational complexity. For long RNAs, however, there remains another problem: numerical overflow. As the standard method for avoiding overflow in stochastic models, logsumexp (logarithm of the sum of exponentials) is not directly applicable to Fourier transforms, we have developed an extended logsumexp method for whole complex numbers. We have shown that reduced computational time enables us to analyze the thermal stability of long RNAs, such as 16S ribosomal RNAs, while the predicted structures using the same maximum-span constraint tend to be inaccurate.

We have also adopted accuracy-guaranteed numerical computation with interval arithmetic to evaluate numerical errors. We have shown that numerical errors for small probabilities are substantial when FFT or DFT is used. Quantitative assessment of the observed numerical instabilities, however, revealed that our method achieves sufficient numerical accuracy for thermodynamic stability analysis of RNA secondary structures. These results demonstrate that our method is a powerful tool for understanding long RNAs.

## Methods

### RintW + maximum-span

Initially, we introduced a maximum-span constraint in base-pairs to the baseline algorithm of RintW [7]. Detailed descriptions of RintW and the proposed method are described below. The inputs of the algorithm are an RNA sequence and a reference secondary structure, and the outputs are the existence probability and the base-pairing probability matrix (BPPM) for each Hamming distance from the reference secondary structure.

#### RNA secondary structure representation

As a computationally efficient expression, the RNA secondary structure was represented by a binary upper triangular matrix *σ* where each element is {0,1}. Each element of *σ* is decided as follows.

$$\sigma_{i,j} = \left\{\begin{array}{ll} 1 & (i < j\; and\; (i,j)\; forms\; a\; base\; pair)\\ 0 & (otherwise)\\ \end{array}\right. $$

The distance between two RNA secondary structures *σ*_1_,*σ*_2_ are determined by the number of elements with different values, namely, Hamming distance values.

We used Hamming distance as the discrete metric of our implementation, as was used in previous studies [2, 5, 7]. The Hamming distance corresponds to the number of unit changes of the secondary structure over time, that is, the forming or breaking a base-pair. Natural distance satisfying axioms can be used to track the trajectory of the structural changes of RNAs. Hamming distance is compatible with efficient dynamic programming algorithms that can be constructed. We know that there are more important base-pairs and less important base-pairs for the function of RNAs, but Hamming distance is at least a convenient metric to observe the landscape of the probability distribution of the secondary structures.

Only secondary structures that satisfy the following constraints were considered (*N*= sequence length).

1. Only Watson–Crick base-pairs (A-T, C-G) or wobble base-pairs(G-U) exist.
2. Prohibition of pseudo-knots: For all 1≤*i*<*j*<*k*<*l*≤*N*,(*i*,*k*) and (*j*,*l*) do not form base-pairs at the same time.
3. Max loop constraint: For all 1≤*i*<*j*<*k*<*l*≤*N*, if (*i*,*l*) and (*j*,*k*) form base-pairs and no paired base exists between *i*+1 and *j*−1 nor between *k*+1 and *l*−1, then *j*−*i*+*l*−*k*≤*C*+2. *C* is a max-loop parameter, and we used *C*=30 following many previous studies.
4. Max span constraint: For all 1≤*i*<*j*≤*N*, if (*i*,*j*) forms a base-pair, then *j*−*i*≤*W*.

Constraints 1 and 2 are the standard constraints used in the previous methods [2, 5, 7]. Constraint 3 is called the max loop constraint. This constraint was adopted by many RNA secondary structure analysis methods using the energy model [14] described in the next section. This constraint reduces time complexity. It is empirically known that this constraint has little effect on the calculation result. Constraint 4 is a constraint studied in previous work [12, 13, 23–25], but it was not used in RintW[7] until we introduced it. This constraint is considered to be suitable for examining local structural motifs [13].

#### Energy model

The nearest neighbor energy model [14], which can be analyzed by dynamic programming, was adopted. The energy of the secondary structure was expressed as the sum of the following five functions in this model.

1. *f*_*h*_(*i*,*j*)= the energy of base-pair (*i*,*j*) forming a hairpin loop.
2. *f*_*l*_(*i*,*j*,*k*,*l*)= the energy of base-pairs, (*i*,*l*) and (*j*,*k*), making a 2-Loop when *i*<*j*<*k*<*l*.
3. *f*_*mc*_= the energy of having one multi-loop.
4. *f*_*mi*_= the energy of having one internal multi-loop branch.
5. *f*_*d*_(*i*,*j*)= the energy of a base-pair (*i*,*j*) forming a multi-loop or being an outermost base-pair.

#### Polynomial approach

In previous research [5–7], the polynomial approach was used as a method to reduce the time complexity of dynamic programming. A naïve dynamic programming method requires a convolution operation. This operation is regarded as computation in the spatial domain and is expressed by calculation in the frequency domain. A convolution operation can be converted to an inner product, thus reducing computational complexity. After completing the dynamic programming computation, a shift to the spatial domain is achieved by performing the Fourier transform. The same method was used in this study.

#### Preprocessing

As in the RintW algorithm, we calculate the following $g_{0}^{Z}(i,j)$ functions in *O*(*N*^2^) time as preprocessing, to obtain the gains of the Hamming distances $g^{Z}_{1}$ to $g^{Z}_{8}$ and $g^{W}_{1}$ to $g^{W}_{5}$:

for (1≤*i*≤*N*)

$$g_{0}^{Z}(i,i) =g_{0}^{Z}(i,i-1)=0 $$

for (1≤*i*<*j*≤*N*)

$$\begin{aligned} & g_{0}^{Z}(i,j) =\sum_{p=i}^{j-1}\sum_{q=p+1}^{j}\sigma_{pq}\\ & =\sigma_{ij}+g_{0}^{Z}(i+1,j)+g_{0}^{Z}(i,j-1)-g_{0}^{Z}(i+1,j-1). \end{aligned} $$

Here, *σ* is a binary matrix representation of the reference secondary structure. The maximum Hamming distance of the secondary structure from the representative secondary structure (*H*_*max*_) is also computed at this time [6].

#### Definitions of function *g*s

Prior to the description of the main algorithm, auxiliary functions for calculating the distance between substructures are defined as follows. These functions are the same as those used in previous studies [6, 7].

$$\begin{aligned} b(i,j)&=1-2\sigma_{ij}\\ g_{1}^{Z}(i,j)&=g_{0}^{Z}(i,j)\\ g_{2}^{Z}(i,j,k)&=g_{0}^{Z}(i,j)-g_{0}^{Z}(i,k)-g_{0}^{Z}(k+1,j)\\ g_{3}^{Z}(i,j,k)&=g_{0}^{Z}(i,j)-g_{0}^{Z}(i,k)\\ g_{4}^{Z}(i,j)&=g_{0}^{Z}(i,j)+b(i,j)\\ g_{5}^{Z}(i,j,k,l)&=g_{0}^{Z}(i,j)-g_{0}^{Z}(k,l)+b(i,j)\\ g_{6}^{Z}(i,j,k)&=g_{0}^{Z}(i,j)-g_{0}^{Z}(i+1,j-1)-g_{0}^{Z}(k,j-1)+b(i,j)\\ g_{7}^{Z}(i,j,k)&=g_{0}^{Z}(i,j)-g_{0}^{Z}(k,j)\\ g_{8}^{Z}(i,j,k)&=g_{0}^{Z}(i,j)-g_{0}^{Z}(i,k-1)-g_{0}^{Z}(k,j)\\ g_{9}^{Z}(i,j,k)&=g_{3}^{Z}(i,j,k)\\ g_{1}^{W}(i,j)&=g_{0}^{Z}(1,N)-g_{0}^{Z}(i,j)-g_{0}^{Z}(1,i-1)-g_{0}^{Z}(j+1,N)\\ g_{2}^{W}(i,j,h,l)&=g_{0}^{Z}(h,l)-g_{0}^{Z}(i,j)+b(h,l)\\ g_{3}^{W}(i,j,h,l)&=g_{2}^{W}(i,j,h,l)-g_{0}^{Z}(h+1,i-1)\\ g_{4}^{W}(i,j,h,l)&=g_{2}^{W}(i,j,h,l)-g_{0}^{Z}(j+1,l-1)\\ g_{5}^{W}(i,j,h,l)&=g_{2}^{W}(i,j,h,l)-g_{0}^{Z}(h+1,i-1)-g_{0}^{Z}(j+1,l-1).\\ \end{aligned} $$

These functions calculate the Hamming distance of a substructure from the reference substructure. More specifically, in the binary matrix representation of the structure, each $g_{*}^{*}$ accumulates differences in rectangular regions of the matrix. According to Mori et al. [6], by changing this function, one can decompose the structures by another distance metric (i.e., other than the Hamming distance), which indicates further potential of this concept, but this was outside of the scope of the study.

#### Dynamic programming of the partition function

In the following equations, *x* is the (*H*_*max*_+1)-th root of unity. If Cooley–Tukey fast Fourier transform (FFT) is used instead of discrete Fourier transform (DFT) in post-processing, *x* is the smallest power of 2 that is equal to or greater than (*H*_*max*_+1). There are (*H*_*max*_+1) kinds of (*H*_*max*_+1)-th roots of unity calculated independently. Therefore, parallel computation is possible.

In order to avoid overflow, the proposed extended logsumexp computation is used. In the following equations, $g^{*}_{*}$ and $-\frac {f_{*}}{kT}$ are real exact numbers (i.e., logsumexp was not applied), but $x^{g^{*}_{*}}$ and $e^{-\frac {f_{*}}{kT}}$ are converted into a complex logsumexp type (i.e., only exponents were recorded). Consequently, All DP-variable $Z^{*}_{*,*}, W^{*}_{*,*}$, and $Q^{*}_{*,*}$ values are also of complex logsumexp type.

Initialization:

$$\begin{aligned} \text{for}\; (1 \leq i &\leq N)\\ Z_{i,i}&=Z_{i,i-1}=1\\ Z_{i,i}^{1}&=Z_{i,i}^{b}=Z_{i,i}^{m}=Z_{i,i-1}^{m}=Z_{i,i}^{m1}=0\\ W_{1,N}^{b} &= \left\{\begin{array}{ll} 1 &((1,N)\; forms\; a\; base\; pair)\\ 0 &(otherwise)\\ \end{array}\right. \end{aligned} $$

Recursion:

for (1≤*i*<*j*≤*N*) s.t. (*j*−*i*≤*W*)

$${\begin{aligned} Z_{1,j}&=x^{g_{1}^{Z}(1,j)}+\sum_{h=1}^{j-1}Z_{1,h-1}Z_{h,j}^{1}x^{g_{2}^{Z}(1,j,h)}\\ Z_{i,N}&=Z_{i+1,N}x^{g_{7}^{Z}(i,N,i+1)}+\\ &\sum_{h=i+1}^{min(N,i+W)}Z_{i,h}^{b}e^{-\frac{f_{d}(i,h)}{kT}}Z_{h+1,N}x^{g_{2}^{Z}(i,N,h)}\\ Z_{i,j}^{1}&=\sum_{h=i+1}^{min(j,i+W)}Z_{i,h}^{b}e^{-\frac{f_{d}(i,h)}{kT}}x^{g_{3}^{Z}(i,j,h)}\\ Z_{i,j}^{b}&=e^{-\frac{f_{h}(i,j)}{kT}}x^{g_{4}^{Z}(i,j)}\\ &+\sum_{h=i+1}^{min(i+C+1,j-2)}\sum_{l=max(h+1, j+h-i-C-2)}^{j-1}Z_{h,l}^{b}e^{-\frac{f_{l}(i,h,l,j)}{kT}}x^{g_{5}^{Z}(i,h,l,j)}\\ &+\sum_{h=i+2}^{j-1}Z_{i+1,h-1}^{m}Z_{h,j-1}^{m1}e^{-\frac{f_{d}(j,i)+f_{mc}}{kT}}x^{g_{6}^{Z}(i,j,h)}\\ Z_{i,j}^{m}&=\sum_{h=i}^{j-1}(x^{g_{7}^{Z}(i,j,h)}+Z_{i,h-1}^{m}x^{g_{8}^{Z}(i,j,h)})Z_{h,j}^{m1}\\ Z_{i,j}^{m1}&=\sum_{h=i+1}^{j}Z_{i,h}^{b}e^{-\frac{f_{d}(i,h)+f_{mi}}{kT}}x^{g_{3}^{Z}(i,j,h)}\\ W_{i,j}^{b}&=Z_{1,i-1}Z_{j+1,N}e^{-\frac{f_{d}(i,j)}{kT}}x^{g_{1}^{W}(i,j)} \end{aligned}} $$

$${\begin{aligned} &+\sum_{h=max(1,i-C-1,i-W)}^{i-1}\sum_{l=j+1}^{min(N,h+W,j+h-i+C+2)}W_{h,l}^{b}e^{-\frac{f_{l}(h,i,j,l)}{kT}}x^{g_{2}^{W}(h,i,j,l)}\\ &+\sum_{h=max(1,i-W)}^{i-1}\sum_{l=j+1}^{min(N,h+W)}W_{h,l}^{b}e^{-\frac{f_{d}(l,h)+f_{mc}+f_{d}(i,j)+f_{mi}}{kT}}(\\ &\quad Z_{h+1,i-1}^{m}x^{g_{3}^{W}(h,i,j,l)}\\ &+Z_{j+1,l-1}^{m}x^{g_{4}^{W}(h,i,j,l)}\\ &+Z_{h+1,i-1}Z_{j+1,l-1}x^{g_{5}^{W}(h,i,j,l)})\\ Q_{i,j}^{b}&=Z_{i,j}^{b}W_{i,j}^{b}\\ \end{aligned}} $$

The *Z*_∗,∗_ functions are the *inside* partition functions, which represent the sums of all the Boltzmann factors in the corresponding sub-sequences. $Z^{1}_{*,*}, Z^{b}_{*,*}, Z^{m}_{*,*}$, and $Z^{m1}_{*,*}$ are the specified partition functions defined in the McCaskill algorithm [1]. $W^{b}_{i,j}$ is the *outside* partition function, which represents the outside of the base-pair (*i*,*j*). The $Q^{b}_{i,j}$ is the conditional partition function, the sum of all the Boltzmann factors when (*i*,*j*) forms a base-pair.

The intuitive meanings of $Z_{*,*}^{*}$ and $W_{*,*}^{b}$ are as follows. $Z_{i,j}^{b}$ ($W_{i,j}^{b}$) is the inside (outside) partition function of partial structure between the *i*-th base and *j*-th base when the *i*-th base and the *j*-th base form a base-pair. $Z_{i,j}^{*}$ is the inside partition function for different conditions. For example, $Z_{i,j}^{1}$ accumulates the cases in which only one outmost base-pair exists, whose 5’ base is the *i*-th base, while $Z_{i,j}^{m}$ and $Z_{i,j}^{m1}$ are considered only for multi-loops.

The values $g^{Z}_{1}$ to $g^{Z}_{8}$ and $g^{W}_{1}$ to $g^{W}_{5}$, which are computed using the pre-computed function $g_{0}^{Z}(i,j)$, are the gains of the Hamming distance for the transitions represented by the recursions of partition functions. The significant difference from RintW is that the recursions of $Z_{1,n}, Z^{1}_{i,j}$, and $W^{b}_{i,j}$ include the maximum-span constraint *W* of base-pairs in their range of the sum. A small improvement in this approach is that only the required edges, namely, *Z*_1,*j*_ and *Z*_*i*,*N*_, are calculated instead of calculating all *Z*_∗,∗_ values. Regarding the maximum-span constraint of base-pairs, the algorithmic concept is equivalent to the calculation of dynamic programming (DP) variables *α*_*Outer*_ and *β*_*Outer*_ in Rfold [12] and ParasoR [13], but the notation of RintW is followed in the above recursions.

#### Fourier transform and post-processing

The conditional partition function on each Hamming distance, $Q_{i,j}^{b}$, is efficiently obtained by Fourier transformation. For all (*i*,*j*), such that (1≤*i*≤*j*≤*N*) and (*j*−*i*≤*W*), a complex number sequence of (*H*_*max*_+1) elements are calculated. Let *Z*(*d*)_1,*N*_ and $Q(d)_{i,j}^{b}$ be the conditional partition functions for a Hamming distance *d* of *Z*_1,*n*_ and $Q_{i,j}^{b}$, respectively. Then, the existence probability of Hamming distance *d* is written as

$$\begin{aligned} \frac{Z(d)_{1,N}}{\sum_{d=0}^{H_{max}}Z(d)_{1,N}}, \end{aligned} $$

and the BPPM for Hamming distance *d* is written as

$$\begin{aligned} \frac{Q(d)_{i,j}^{b}}{Z(d)_{1,N}}. \end{aligned} $$

The obtained partition functions and probabilities mutually differ by several tens of digits. However, since all variables are convoluted during post-processing, all numerical errors propagate to all variables. This makes marginal probabilities of small values unreliable.

#### Computational complexity

In the following description, *N* is the length of the sequence, *H*_*max*_ is the maximum Hamming distance from the reference structure, and *U* is the degree of parallelism. Here *U*≤*H*_*max*_+1 is assumed. In the original RintW algorithm, the computational complexity of pre-processing is *O*(*N*^2^) in both time and space. In the partition function calculation, the time complexity is *O*(*N*^4^*H*_*max*_/*U*) and the space complexity is *O*(*N*^2^*H*_*max*_*U*). The original RintW uses DFT for post-processing; the time complexity of the post-processing part is $O(N^{2}H_{max}^{2}/U)$, and its space complexity is *O*(*H*_*max*_*U*). Since *H*_*max*_≤*N* holds, the computational complexity of the post-processing can be ignored in total complexity in both time and space. Finally, the time and space complexity of the original RintW algorithm as a whole are *O*(*N*^4^*H*_*max*_/*U*) and *O*(*N*^2^*H*_*max*_*U*), respectively.

When the maximum-span constraint is introduced, the computational complexity of pre-processing remains *O*(*N*^2^) in both time and space. In the distribution function calculation, the time complexity is *O*(*NW*^3^*H*_*max*_/*U*), and the space complexity is *O*(*NWH*_*max*_*U*) for the maximum-span of base-pair *W*. When DFT is used for post-processing, the complexity of the post-processing is $O(NWH_{max}^{2}/U)$ in time and *O*(*H*_*max*_*U*) in space. Because the *H*_*max*_ may be close to *N*, the computational complexity of the post-processing cannot be ignored. By using FFT instead of DFT, we can reduce the time complexity of the post-processing component to *O*(*NWH*_*max*_*log*(*H*_*max*_)/*U*). Then, the total computational complexity is *O*(*N*(*N*+*WH*_*max*_(*W*^2^+*log*(*H*_*max*_))/*U*)) in time and *O*(*N*(*N*+*WH*_*max*_*U*)) in space.

The summary of computational complexities is shown, with the notation simplified by using *H*_*max*_≤*N*, in Table 1.

**Table 1:** Computational complexity of the existing and proposed methods are summarized

	RintW, time	RintC (proposed), time
preprocessing	*O*(*N*^2^)	*O*(*N*^2^)
main calculation	*O*(*N*^5^/*U*)	*O*(*N*^2^*W*^3^/*U*)
postprocessing (DFT)	*O*(*N*^4^/*U*)	*O*(*N*^3^*W*/*U*)
postprocessing (FFT)	*O*(*N*^3^*logN*/*U*)	*O*(*N*^2^*WlogN*/*U*)
total (DFT)	*O*(*N*^5^/*U*)	*O*(*N*^2^*W*(*W*^2^+*N*)/*U*)
total (FFT)	*O*(*N*^5^/*U*)	*O*(*N*^2^*W*(*W*^2^+*logN*)/*U*)
	RintW, space	RintC (proposed), space
preprocessing	*O*(*N*^2^)	*O*(*N*^2^)
main calculation	*O*(*N*^3^*U*)	*O*(*N*^2^*WU*)
postprocessing (DFT)	*O*(*NU*)	*O*(*NU*)
postprocessing (FFT)	*O*(*NU*)	*O*(*NU*)
total (DFT)	*O*(*N*^3^*U*)	*O*(*N*^2^*WU*)
total (FFT)	*O*(*N*^3^*U*)	*O*(*N*^2^*WU*)

*N* = sequence length. *W* = maximum-span. Note that *H*_*max*_≤*N* and *W*≤*N* always holds. *U* = degree of parallelism

### Interval arithmetic and accuracy assurance

In this subsection, we briefly explain the rounding mode control function of IEEE 754 and the accuracy assurance arithmetic. Representing real numbers by floating-point numbers can cause deviations from actual values. Therefore, numerical values can conceivably be held as an interval including the actual value. We define arithmetic operations between intervals to obtain an interval necessarily containing the results of arithmetic operations on actual values. Then, the upper bound of the numerical error is obtained as the width of the interval of the calculation result. Most modern computers use the IEEE 754 method for floating-point arithmetic. This method has a rounding mode control function, and we can specify truncation and rounding-up. By using this function, the accuracy assurance calculation described above can be executed efficiently. Our accuracy assurance calculation used the kv library [26]) implemented in C++. The kv library is open source software and requires only C++ Boost for its backend.

### Logsumexp on complex numbers with interval arithmetic

A method to perform logsumexp computation on whole complex numbers has been developed. Details of the calculation algorithm are provided in the following subsections. There are different parts of algorithms for scalar and interval types, but those for scalar types are described in the supplementary file. In this subsection, only methods for interval types are described. If only the scalar type is considered, the complex number defined in polar coordinates and logsumexp defined only in terms of a radius are sufficient. Extensions to interval arithmetic, however, are complicated.

The Vienna RNA Package [27] prevents overflow by scaling. Their scaling factor construction is sophisticated, and under some assumptions, the scaling is equivalent to a kind of logsumexp. The original RintW [7] also utilized the same scaling technique as Vienna. However, with Vienna’s method, the deviation between the scaling factor and the value of the actual distribution function can increase exponentially, so overflowing cannot be completely avoided. Unlike them, logsumexp does not need scaling factors, and overflows are completely avoided.

#### Notation and representation

In this subsection, a bracketing character like [*x*] indicates an interval type variable. A pair of values in a bracket (e.g., [0,1]) indicates a closed interval. When two variables are enclosed (e.g., [*x*,*y*]), each variable *x* and *y* is a scalar type (or floating-point type), not an interval type. It is possible to convert one scalar *x* into an interval type while guaranteeing accuracy. Such an interval variable is expressed as [*x*,*x*] (i.e., [*x*,*x*] is an interval that includes the real value *x*). Finally, a function *f*_*upper*_([*x*])=*u* for obtaining the maximum value of the interval type variable [*x*]=[*l*,*u*], a function *f*_*lower*_([*x*])=*l* for obtaining the minimum value, and a function $f_{mid}([x])=\frac {l+u}{2}$ for obtaining the median value are used. However, it is assumed that they are not necessarily accuracy-guaranteed functions.

To represent the complex number [*a*]+[*b*]*i*,([*r*],[*c*],[*d*]) is held for

$$[a]+[b]i \subseteq e^{[r]}([c]+[d]i) $$.

However, as a normalization condition,

$$ f_{upper}([c]^{2}+[d]^{2}) = \left\{\begin{array}{ll} 0 &([a]=[0,0] \quad and \quad [b]=[0,0])\\ 1 &(otherwise)\\ \end{array}\right. $$

must be satisfied. It is assumed that 1 is numerically almost 1. The difference from 1 accumulates by multiplication, but it is reset by addition. For convenience, [*r*]=[0,0] must be satisfied when ([*a*]=[0,0] *and* [*b*]=[0,0]).

The conversion protocol between this and the usual representation is described in the supplementary file. Normalization, multiplication, and addition protocols are described below.

#### Normalization

When a number ([*r*^′^],[*c*^′^],[*d*^′^]) that is not normalized is given, a method of obtaining the normalized number with accuracy assurance ([*r*],[*c*],[*d*])⊇([*r*^′^],[*c*^′^],[*d*^′^]) is as follows.


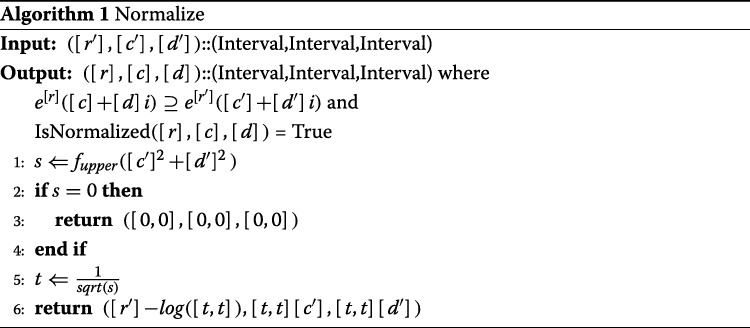


Description:

First, compute

$$\begin{aligned} s &= f_{upper}([c']^{2}+[d']^{2})\\ t &= \frac{1}{sqrt(s)}\\, \end{aligned} $$

where *t* is the reciprocal of the maximum value of the absolute value of the input. At this time

$$ ([r],[c],[d]) = \left\{\begin{array}{ll} ([0,0],[0,0],[0,0]) & (s=0)\\ ([r']-log([t,t]),[t,t][c'],[t,t][d']) & (otherwise)\\ \end{array}\right. $$

is a normalized solution.

#### Multiplication

The multiplication of the two values ([*r*_1_],[*c*_1_],[*d*_1_]) and ([*r*_2_],[*c*_2_],[*d*_2_]) can be described as

$$\begin{aligned} &([r_{1}],[c_{1}],[d_{1}])([r_{2}],[c_{2}],[d_{2}])\\ &= e^{[r_{1}]}([c_{1}]+[d_{1}]i)e^{[r_{2}]}([c_{2}]+[d_{2}]i)\\ &= e^{[r_{1}]+[r_{2}]}([c_{1}]+[d_{1}]i)([c_{2}]+[d_{2}]i)\\ &= e^{[r_{1}]+[r_{2}]}(([c_{1}][c_{2}]-[d_{1}][d_{2}])+([c_{1}][d_{2}]+[d_{1}][c_{2}])i), \end{aligned} $$

and ([*r*_1_]+[*r*_2_],[*c*_1_][*c*_2_]−[*d*_1_][*d*_2_],[*c*_1_][*d*_2_]+[*d*_1_][*c*_2_]) is obtained as a solution. In normalization post-processing, if [*c*_1_][*c*_2_]−[*d*_1_][*d*_2_]=[*c*_1_][*d*_2_]+[*d*_1_][*c*_2_]=[0,0],[*r*]=[0,0] is substituted. Otherwise, because the product of the complex numbers with absolute value 1 is absolute value 1, it is naturally normalized.


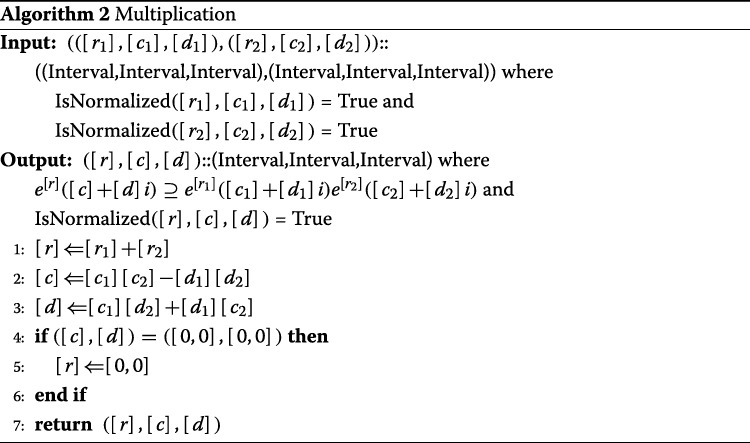


#### Addition

Consider the sum of the two values ([*r*_1_],[*c*_1_],[*d*_1_]) and ([*r*_2_],[*c*_2_],[*d*_2_]). As addition is commutative, assuming *f*_*mid*_([*r*_1_])≥*f*_*mid*_([*r*_2_]) does not decrease generality. Then, it can be formulated as

$$\begin{aligned} p &= f_{upper}([r_{1}])-f_{mid}([r_{1}])\\ &+f_{upper}([r_{2}])-f_{mid}([r_{2}]) \quad (p \geq 0)\\ \end{aligned} $$

and

$$\begin{aligned} & ([r_{1}],[c_{1}],[d_{1}])+([r_{2}],[c_{2}],[d_{2}])\\ &=e^{[r_{1}]}([c_{1}]+[d_{1}]i)+e^{[r_{2}]}([c_{2}]+[d_{2}]i)\\ &=e^{[r_{1}]}([c_{1}]+[d_{1}]i)+e^{[r_{1}]}(e^{[r_{2}]-[r_{1}]}[c_{2}]+e^{[r_{2}]-[r_{1}]}[d_{2}]i)\\ &=e^{[r_{1}]}(([c_{1}]+e^{[r_{2}]-[r_{1}]}[c_{2}])+([d_{1}]+e^{[r_{2}]-[r_{1}]}[d_{2}])i)\\ &=e^{[r_{1}]+[p,p]}((e^{-[p,p]}[c_{1}]+e^{[r_{2}]-[r_{1}]-[p,p]}[c_{2}])\\ &\quad +(e^{-[p,p]}[d_{1}]+e^{[r_{2}]-[r_{1}]-[p,p]}[d_{2}])i). \end{aligned} $$

Thus, *f*_*upper*_(*e*^−[*p*,*p*]^)≤1 follows from the assumption of *p*≥0. Additionally, $\phantom {\dot {i}\!}f_{upper}(e^{[r_{2}]-[r_{1}]-[p,p]}) \leq 1$ follows from the assumption that *f*_*mid*_([*r*_1_])≥*f*_*mid*_([*r*_2_]) (the proof is provided in the supplementary file). Therefore, *e*^−[*p*,*p*]^ and $\phantom {\dot {i}\!}e^{[r_{2}]-[r_{1}]-[p,p]}$ can be directly calculated without overflow occurring. Therefore,

$$\begin{aligned} [r']&=[r_{1}]+[p,p]\\ [c']&=(e^{-[p,p]}[c_{1}]+e^{[r_{2}]-[r_{1}]-[p,p]}[c_{2}])\\ [d']&=(e^{-[p,p]}[d_{1}]+e^{[r_{2}]-[r_{1}]-[p,p]}[d_{2}])\\ \end{aligned} $$

can be calculated, and ([*r*^′^],[*c*^′^],[*d*^′^]) satisfies

$$\begin{aligned} ([r_{1}],[c_{1}],[d_{1}])+([r_{2}],[c_{2}],[d_{2}]) &= ([r'],[c'],[d'])\\ \end{aligned} $$

as the summation. Finally, since this is not normalized, normalization processing is required.


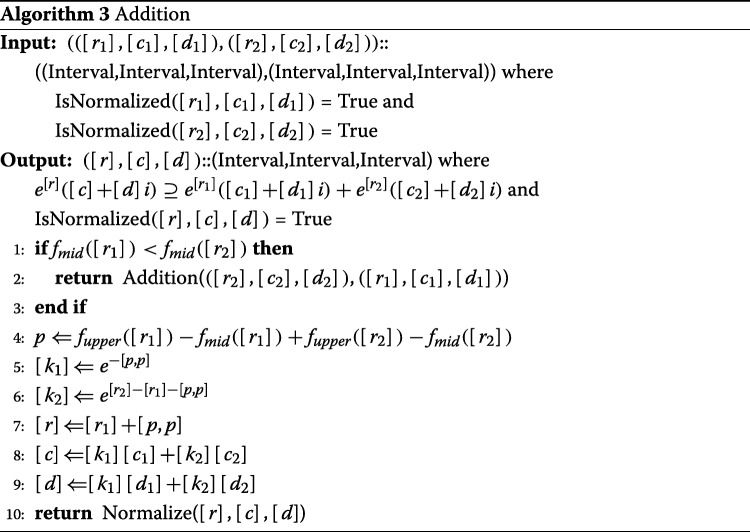


In the classic logsumexp, numerical errors of summation are reduced by using a summation-specific technique rather than recursively using the two-operand addition function. For the summation-specific technique, in three or more operands, one can use the maximum number as the scaling factor and scale the others. On the other hand, we developed only the normal two-operand addition function. The following experiment shows that our method brings sufficient numerical accuracy. Nevertheless, further improvement may still be possible.

#### Requirements for an accuracy assurance calculation library

The functions that the accuracy assurance calculation library must perform in this method are as follows:

1. The conversion from scalar to interval type guarantees accuracy.
2. Four arithmetic operations, log, and exp, with accuracy assurance for the interval type.
3. The previously described *f*_*upper*_([*x*]) and *f*_*mid*_([*x*]).

### Credibility limit

In order to evaluate the magnitude of thermal fluctuation, we used Credibility Limit [18] as the metric. Credibility Limit is the minimum distance in which a certain percentage of structures is distributed. More specifically, given a representative structure *σ* and a distance *d*, consider *p*_*d*_, the sum of Boltzmann probabilities of structures whose distances from *σ* are less than or equal to *d*. Then, given a probability value *p*, CL(p) is the smallest *d* such that *p*_*d*_≥*p*. The larger the Credibility Limit value, the more intense the thermal fluctuation of the molecule.

### Experimental procedure

The S151 Rfam Dataset ‘with all pseudoknots removed’ [15] was used for evaluation of time complexity and numerical accuracy in interval operation.

For the application of our proposed method to RNA molecules longer than those in the S151 Rfam dataset [15], the primary sequences and the corresponding native secondary structures of 16S rRNAs were obtained from three-dimensional structures of *E. coli* and *T. thermophilus*, while those of 70S ribosomes were from the Nucleic Acid Database (NDB) [28, 29]. The NDB IDs of the *E. coli* and *T. thermophilus* ribosome structures were 4V9D (chainID: AA) [30] and 4V51 (chainID: AA) [31], respectively. As the secondary structures of these 16S rRNAs, base-pairs were selected according to the "base-pair hydrogen bonding classification" provided by NDB. Specifically, base-pairs were classified as 1 in the Leontis–Westhof classification [32] and either 19, 20, or 28 in the Saenger classification [33]. Base-to-base correspondence between the primary sequence and its secondary structure (derived from the three-dimensional structure in which several residues are missing) was estimated using Needleman–Wunsch alignment [34].

The energy parameter rna_turner2004.par included in the Vienna RNA package [27] version 2.4.9 was used. However, the source code itself of Vienna was not used. The algorithms were implemented by the authors, except for parameter file reading, which is based on ParasoR’s implementation [13].

## Supplementary information

**Additional file 1** Supplementary PDF file.

## Data Availability

The source code for RintC is available on the following website. https://github.com/eukaryo/rintc The S151Rfam dataset is available on the following website. http://contra.stanford.edu/contrafold/download.html

## Abbreviations

BPPM: Base-pairing probability matrix
DFT: Discrete fourier transform
DP: Dynamic programming
FFT: Fast fourier transform
NDB: Nucleic acid database
ncRNA: Non-coding RNA
rRNA: Ribosomal RNA
