# Supplementary material for: RintC: fast and accuracy-aware decomposition of distributions of RNA secondary structures with extended logsumexp
Source: BMC Bioinformatics. 2020 May 24;21:210. doi: 10.1186/s12859-020-3535-5 (PMC7245837; doi:10.1186/s12859-020-3535-5)
Supplement: Supplementary file 1 — Additional file 1 Supplementary PDF file. [file 12859_2020_3535_MOESM1_ESM.pdf]

Supplementary file for RintC: ultra-fast and accuracy-aware decomposition of distribution on RNA secondary structures

Hiroki Takizawa, Junichi Iwakiri, and Kiyoshi Asai

October 9, 2019

1 All Functions for Logsumexp on Complex Number but not Interval

Here we explain how to perform logsumexp on the whole complex number. In this subsection, we describe methods for the normal scalar type (or floating point number type) which is not an interval type. In this section, the base of log is e.

1.1 representation

First, as a representation of the complex number $a + bi$, hold (r, c, d) of

$$a + bi = e^r(c + di)$$

However, as a normalization condition,

$$c^2 + d^2 = \begin{cases} 0 & (a + bi = 0) \\ 1 & (otherwise) \end{cases}$$

must be satisfied. For convenience, it must be satisfied that $r = 0$ when $a + bi = 0$. The algorithm for checking whether (r, c, d) is normalized can be written as follows.

Algorithm 1 IsNormalized

Input: (r, c, d)::(Real,Real,Real)
Output: Bool

if $(c, d) = (0, 0)$ **then**
  if $r = 0$ **then**
    return True
  end if
  return False
end if
if $c^2 + d^2 = 1$ **then**
  return True
end if
return False

1.2 normalization

When a number (r', c', d') which is not normalized is given, a method of obtaining the normalized number $(r, c, d) = (r', c', d')$ is as follows.

Algorithm 2 Normalize

Input: (r', c', d')::(Real,Real,Real)
Output: (r, c, d)::(Real,Real,Real) where
  $e^r(c + di) = e^{r'}(c' + d'i)$ and IsNormalized(r, c, d) = True

$s \Leftarrow (c')^2 + (d')^2$
if $s = 0$ **then**
  return $(0, 0, 0)$
end if
$t \Leftarrow \frac{1}{sqrt(s)}$
return $(r' - log(t), tc', td')$

Description:
First, compute

$$s = (c')^2 + (d')^2$$
$$t = \frac{1}{sqrt(s)}$$

t is the reciprocal of the absolute value of the input. At this time

$$(r, c, d) = \begin{cases} (0, 0, 0) & (s = 0) \\ (r' - log(t), tc', td') & (otherwise) \end{cases}$$

is a normalized solution.

1.3 conversion

To obtain (r, c, d) when (a, b) such as $a + bi$ is given, normalize $(0, a, b)$.

Algorithm 3 ConvertUsualIntoWide

Input: (a, b)::(Real,Real)
Output: (r, c, d)::(Real,Real,Real) where
$e^r(c + di) = (a + bi)$ and IsNormalized(r, c, d) = True

return Normalize$(0, a, b)$

For inverse transformation, calculate normally.

Algorithm 4 ConvertWideIntoUsual

Input: (r, c, d)::(Real,Real,Real)
Output: (a, b)::(Real,Real) where $(a + bi) = e^r(c + di)$

return $(e^r c, e^r d)$

1.4 multiplication

Algorithm 5 Multiplication

Input: $((r_1, c_1, d_1), (r_2, c_2, d_2))$::
((Real,Real,Real),(Real,Real,Real)) where
IsNormalized(r_1, c_1, d_1) = True and
IsNormalized(r_2, c_2, d_2) = True
Output: (r, c, d)::(Real,Real,Real) where
$e^r(c + di) = e^{r_1}(c_1 + d_1 i)e^{r_2}(c_2 + d_2 i)$ and
IsNormalized(r, c, d) = True

$r \Leftarrow r_1 + r_2$
$c \Leftarrow c_1 c_2 - d_1 d_2$
$d \Leftarrow c_1 d_2 + d_1 c_2$
if $(c, d) = (0, 0)$ **then**
  $r \Leftarrow 0$
end if
return (r, c, d)

Description:
The multiplication of the two values (r_1, c_1, d_1) and (r_2, c_2, d_2) can be descripted as

$$\begin{aligned}
&(r_1, c_1, d_1)(r_2, c_2, d_2) \\
&= e^{r_1}(c_1 + d_1 i)e^{r_2}(c_2 + d_2 i) \\
&= e^{r_1 + r_2}(c_1 + d_1 i)(c_2 + d_2 i) \\
&= e^{r_1 + r_2}((c_1 c_2 - d_1 d_2) + (c_1 d_2 + d_1 c_2)i)
\end{aligned}$$

and $(r_1 + r_2, c_1c_2 - d_1d_2, c_1d_2 + d_1c_2)$ is obtained as a solution. As a post-processing for normalization, if $c_1c_2 - d_1d_2 = c_1d_2 + d_1c_2 = 0$, substitute $r = 0$. Otherwise, since the product of the complex numbers with absolute value 1 is absolute value 1, it is naturally normalized.

1.5 addition

Algorithm 6 Addition

Input: $((r_1, c_1, d_1), (r_2, c_2, d_2))$::
  ((Real,Real,Real),(Real,Real,Real)) where
    IsNormalized(r_1, c_1, d_1) = True and
    IsNormalized(r_2, c_2, d_2) = True

Output: (r, c, d)::(Real,Real,Real) where
  $e^r(c + di) = e^{r_1}(c_1 + d_1i) + e^{r_2}(c_2 + d_2i)$ and
  IsNormalized(r, c, d) = True

if $r_1 < r_2$ **then**
  return Addition$((r_2, c_2, d_2), (r_1, c_1, d_1))$
end if
$k \Leftarrow e^{r_2 - r_1}$
$r \Leftarrow r_1$
$c \Leftarrow c_1 + kc_2$
$d \Leftarrow d_1 + kd_2$
return Normalize(r, c, d)

Description:

Consider adding the two values (r_1, c_1, d_1) and (r_2, c_2, d_2). Since addition is commutative, assuming $r_1 \geq r_2$ does not lose generality. Then, it can be formulated as

$$\begin{aligned}
&(r_1, c_1, d_1) + (r_2, c_2, d_2) \\
&= e^{r_1}(c_1 + d_1i) + e^{r_2}(c_2 + d_2i) \\
&= e^{r_1}(c_1 + d_1i) + e^{r_1}(e^{r_2 - r_1}c_2 + e^{r_2 - r_1}d_2i) \\
&= e^{r_1}((c_1 + e^{r_2 - r_1}c_2) + (d_1 + e^{r_2 - r_1}d_2)i)
\end{aligned}$$

Since it is $e^{r_2 - r_1} \leq 1$ from the assumption of $r_1 \geq r_2$, $e^{r_2 - r_1}$ can be directly calculated without overflowing. Therefore,

$$\begin{aligned}
c' &= (c_1 + e^{r_2 - r_1}c_2) \\
d' &= (d_1 + e^{r_2 - r_1}d_2)
\end{aligned}$$

can be calculated and (r_1, c', d') satisfies

$$(r_1, c_1, d_1) + (r_2, c_2, d_2) = (r_1, c', d')$$

as an answer of addition. Finally, since this is not normalized, it needs normalization processing.

2 Auxilary Functions for Logsumexp on Complex Number and Interval

The algorithm for checking whether $([r],[c],[d])$ is normalized can be written as follows.

Algorithm 7 IsNormalized

Input: $([r],[c],[d])$::(Interval,Interval,Interval)
Output: Bool

if $(c,d) = ([0,0],[0,0])$ **then**
  if $r = [0,0]$ **then**
    return True
  end if
  return False
end if
if $f_{upper}(c^2 + d^2) \approx 1$ **then**
  return True
end if
return False

2.1 conversion

To obtain $([r],[c],[d])$ when $[a]+[b]i$ is given, normalize $([0,0],[a],[b])$.For inverse transformation, calculate normally.

Algorithm 8 ConvertUsualIntoWide

Input: $([a],[b])$::(Interval,Interval)
Output: $([r],[c],[d])$::(Interval,Interval,Interval) where $e^{[r]}([c]+[d]i) \supseteq ([a]+[b]i)$ and IsNormalized$([r],[c],[d])$ = True

return Normalize$([0,0],[a],[b])$

Algorithm 9 ConvertWideIntoUsual

Input: $([r],[c],[d])$::(Interval,Interval,Interval)
Output: $([a],[b])$::(Interval,Interval) where $([a]+[b]i) \supseteq e^{[r]}([c]+[d]i)$

return $(e^{[r]}[c], e^{[r]}[d])$

3 Proof of Proposition 1

Proposition 1: $f_{upper}(e^{[r_2]-[r_1]-[p,p]}) \leq 1$

Proof:

Since they are assumptions with $f_{mid}([r_1]) \geq f_{mid}([r_2])$ and

$$p = f_{upper}([r_1]) - f_{mid}([r_1]) \\ + f_{upper}([r_2]) - f_{mid}([r_2]) \quad (p \geq 0)$$

, it holds that

$$\begin{aligned} & f_{upper}([r_2] - [r_1]) \\ & = f_{upper}([r_2]) - f_{lower}([r_1]) \\ & = (f_{upper}([r_2]) - f_{mid}([r_2]) + f_{mid}([r_2])) \\ & \quad - (f_{lower}([r_1]) - f_{mid}([r_1]) + f_{mid}([r_1])) \\ & = (f_{upper}([r_2]) - f_{mid}([r_2]) + f_{mid}([r_2])) \\ & \quad - (-f_{upper}([r_1]) + f_{mid}([r_1]) + f_{mid}([r_1])) \\ & = (f_{upper}([r_1]) - f_{mid}([r_1]) + f_{upper}([r_2]) \\ & \quad - f_{mid}([r_2])) + f_{mid}([r_2]) - f_{mid}([r_1]) \\ & = p + f_{mid}([r_2]) - f_{mid}([r_1]) \\ & \leq p. \end{aligned}$$

Therefore,

$$f_{upper}([r_2] - [r_1] - [p,p]) \leq 0$$

holds. Since,

$$f_{upper}(e^{[r_2]-[r_1]-[p,p]}) \leq 1.$$

QED.
